# Simultaneous Bayesian Estimation of Excitatory and Inhibitory Synaptic Conductances by Exploiting Multiple Recorded Trials

**DOI:** 10.3389/fncom.2016.00110

**Published:** 2016-11-04

**Authors:** Milad Lankarany, Jaime E. Heiss, Ilan Lampl, Taro Toyoizumi

**Affiliations:** ^1^Neurosciences and Mental Health, Department of Physiology and the Institute of Biomaterials and Biomedical Engineering, University of Toronto, The Hospital for Sick ChildrenToronto, ON, Canada; ^2^RIKEN Brain Science InstituteSaitama, Japan; ^3^Center for Neuroscience, Biosciences Division, SRI InternationalMenlo Park, CA, USA; ^4^Department of Neurobiology, Weizmann Institute of ScienceRehovot, Israel

**Keywords:** excitatory and inhibitory synaptic inputs, synaptic conductance, Kalman filter, current clamp, barrel cortex, *in vivo*

## Abstract

Advanced statistical methods have enabled trial-by-trial inference of the underlying excitatory and inhibitory synaptic conductances (SCs) of membrane-potential recordings. Simultaneous inference of both excitatory and inhibitory SCs sheds light on the neural circuits underlying the neural activity and advances our understanding of neural information processing. Conventional Bayesian methods can infer excitatory and inhibitory SCs based on a single trial of observed membrane potential. However, if multiple recorded trials are available, this typically leads to suboptimal estimation because they neglect common statistics (of synaptic inputs (SIs)) across trials. Here, we establish a new expectation maximization (EM) algorithm that improves such single-trial Bayesian methods by exploiting multiple recorded trials to extract common SI statistics across the trials. In this paper, the proposed EM algorithm is embedded in parallel Kalman filters or particle filters for multiple recorded trials to integrate their outputs to iteratively update the common SI statistics. These statistics are then used to infer the excitatory and inhibitory SCs of individual trials. We demonstrate the superior performance of multiple-trial Kalman filtering (MtKF) and particle filtering (MtPF) relative to that of the corresponding single-trial methods. While relative estimation error of excitatory and inhibitory SCs is known to depend on the level of current injection into a cell, our numerical simulations using MtKF show that both excitatory and inhibitory SCs are reliably inferred using an optimal level of current injection. Finally, we validate the robustness and applicability of our technique through simulation studies, and we apply MtKF to *in vivo* data recorded from the rat barrel cortex.

## Introduction

Inferring the excitatory and inhibitory synaptic conductances (SCs) from single trials of membrane-potential recordings, and their underlying trial-to-trial variability, is crucial for understanding various functional aspects of neuronal sensory response, such as the neuron's receptive fields (Anderson et al., 2001; Wehr and Zador, 2003; Priebe and Ferster, 2005) or unveiling the mechanisms of adaptation (Katz et al., 2006; Heiss et al., 2008; Ramirez et al., 2014). The importance of such variability in understanding the neuronal mechanisms of brain activity and their key roles in information processing is well reviewed in Destexhe and Contreras (2006). A standard approach to inferring these conductances is first to average the membrane potential (MP) (or current, if cells are recorded under voltage clamp) responses triggered by a stereotypic external event, such as sensory stimulation, for each level of holding current. Linear regression is then applied to infer how these average responses depend on different holding currents (Shu et al., 2003; Wehr and Zador, 2003; Priebe and Ferster, 2005). However, this analysis provides only the average SCs, ignoring their trial-to-trial variability. While the voltage-clamp technique (Zhang et al., 2003; Murphy and Rieke, 2006; Haider et al., 2013) can yield either excitatory or inhibitory SCs in each trial by setting the holding potential equal to the inhibitory or excitatory reversal potential, respectively, simultaneous recording of both SCs is not possible.

Bédard et al. (2012) proposed a new method based on oversampling the sub-threshold MP to estimate the time course of excitatory and inhibitory SCs from a single recorded voltage trace. Although, this method works well when these conductances change slowly relative to MP, the long predefined oversampling (roughly 20 successive time points) for avoiding algorithmic instability tends to reduce the temporal precision of the estimated SCs. Berg and Ditlevsen (2013) divided the MP into slowly and rapidly varying parts according to a pre-set criterion, and used the autocorrelation function of the rapidly varying part to infer the total conductance. Excitatory and inhibitory SCs were then inferred from the slowly varying part of the MP and the estimated total conductance. This method resulted in accurate estimates of both conductances if the MP could be well approximated as an Ornstein–Uhlenbeck (OU) process, and if the autocorrelation function of the rapidly varying part decayed appreciably within the pre-set window. While these methods are successful in estimating SCs, they do not explicitly model their underlying probability or infer them at the temporal resolution of observation steps. In addition, care should be taken over the dependency of these methods on certain parameter settings, e.g., the oversampling period (Bédard et al., 2012) or the pre-set window (Berg and Ditlevsen, 2013) during which SCs are assumed constant, for achieving optimal estimates in the presence of noise.

Recently, Bayesian-based algorithms (Paninski et al., 2012; Lankarany et al., 2013b) have been derived for inferring excitatory and inhibitory SCs in a more principled manner. In contrast to the above-mentioned statistical approaches, these Bayesian approaches model the probability of SCs at the temporal resolution of observation steps and estimate parameters optimally from data. Paninski et al. (2012) reported promising results for low-level observation noise. They derived a sequential Monte-Carlo method (particle filtering (PF)) for filtering/smoothing the dynamics of a compact neuronal model, and used an expectation maximization (EM) algorithm (in both parametric and non-parametric manners) to infer the time-varying mean of the SCs. Lankarany et al. (2013b) proposed a recursive algorithm based on Gaussian-mixture Kalman filtering for filtering/smoothing the dynamics of a compact neuronal model (the same as used in Paninski et al., 2012). This was followed by an EM algorithm for inferring the statistical parameters of the excitatory and inhibitory synaptic inputs (SIs) using a non-parametric spline method. This methodology provided more degrees of freedom for these inputs by estimating their distributions with a Gaussian mixture model. It is shown in Lankarany et al. (2013b) that this algorithm can be faster and easier than PF in several cases. In both algorithms, a non-parametric EM algorithm is used to estimate time-varying statistics (mean and variance) of excitatory and inhibitory SIs from a single trace of a given MP. However, rapid fluctuations of SCs cannot be reliably estimated. Therefore, the EM algorithm estimates a temporally smooth version of these statistics.

In this paper, we propose a new EM algorithm that improves such single-trial Bayesian methods by exploiting multiple recorded trials to extract the statistics of common SIs across the trials. Our algorithm, multiple-trial Kalman filtering (MtKF), also estimates excitatory and inhibitory SCs from single trials of the sub-threshold MP recorded in response to repeated sensory stimulation. However, unlike previous algorithms, our proposed recursive EM algorithm uses all repeatedly recorded trials of the MP to better estimate the common time-varying statistics of the excitatory and inhibitory SIs. It does this without requiring any parametric or non-parametric smoothing step that could blur the fine temporal features of the conductances. Our recursive algorithm consists of two steps. Firstly, the Kalman filtering (KF) algorithm estimates excitatory and inhibitory SCs from each membrane-potential trace, assuming certain statistics underlying the excitatory and inhibitory SCs. Secondly, these statistics are updated based on all estimated values of excitatory and inhibitory SCs from different trials.

Our simulations demonstrate the accuracy and robustness of our proposed MtKF approach compared to the single-trial KF (StKF) (Lankarany et al., 2013b). Moreover, we show that the multiple-trial framework can be generalized to other single-trial Bayesian methods. We also develop multiple-trial particle filtering (MtPF) and show that it outperforms the single-trial PF (StPF) (Paninski et al., 2012). Moreover, by performing a comprehensive simulation study, we investigate the level of injected current that results in the most accurate simultaneous estimate of both excitatory and inhibitory SCs. We show that the optimum level of current injection must hold the average MP at approximately −50 mV, and this level of MP is almost independent of observation noise level. Applying our algorithm at the best level of injected current constitutes an easier current-clamp recording protocol (i.e., requiring only one level of injected current) for simultaneously inferring both excitatory and inhibitory SCs in single trials at high accuracy.

## Materials and methods

### *In vivo* data

The *in vivo* data was recorded in Ilan Lampl's laboratory at the Weizmann Institute of Science, Rehovot, Israel (*in vivo* whole-cell patch-clamp recording in the rat barrel cortex). All details about the recordings, pre-processing of data, and animal preparations have been reported previously (Heiss et al., 2008). All recordings were made from young (4–7 weeks old) adult Wistar rats. Whole-cell patch recordings were performed in current-clamp mode in the presence of the sodium channel blocker QX-314 (2 mM). The whisker chosen for stimulation was done so using a piezoelectric device. The stimulation velocity and the corresponding deflection amplitude were adjusted to evoke clear sub-threshold responses in the cortical cells located in either L4 or L2/3. Each cell was stimulated by a train of 20 whisker deflections at either 10 or 18 Hz. After initial anesthesia with ketamine (100 mg/kg) and acepromazine maleate (1 mg/kg), a tracheotomy was performed after a local subcutaneous injection of lidocaine. All surgical and experimental procedures were conducted in accordance with the regulations of the Weizmann Institute Animal Care and Use Committee.

### Neuron model

We use a passive neuronal model (Paninski et al., 2012; Lankarany et al., 2013b) that represents the MP dynamics of a single neuron that is receiving SIs. This model, which neglects the active ion channels (but see Vich and Guillamon, 2015, which studies the influence of active channels during subthreshold activity, and (Lankarany et al., 2013a), which infers the parameters of active ion channels), can be expressed as follows (Koch, 1999; Huys et al., 2006; Paninski et al., 2012):

(1) $$\{\begin{array}{l} V(t+1)=V(t)+dt[g_{L}(E_{L}-V(t))+g_{E}(t)(E_{E}-V(t))\\ \;+g_{I}(t)(E_{I}-V(t))]+w(t)\\ g_{E}(t+1)=g_{E}(t)-dt\frac{g_{E}(t)}{\tau_{E}}+N_{E}(t)\\ g_{I}(t+1)=g_{I}(t)-dt\frac{g_{I}(t)}{\tau_{I}}+N_{I}(t) \end{array}$$

Here, *V, g*_*E*_, and *g*_*I*_ are the dynamic variables of the neuron, indicating the MP and excitatory and inhibitory SCs, respectively. The term *w*(*t*) is white Gaussian noise of variance $\sigma_{w}^{2}$, *N*_*E*_(*t*), and *N*_*I*_(*t*) are the instantaneous excitatory and inhibitory SIs to the neuron at time step *t*, respectively, and *dt* is the time bin, which may differ from the voltage recording sampling. Equation (1) is the first-order (Euler method) approximation of the underlying continuous-time differential equation. Note that the time index *t* takes integer values between *0* and *T*, where *T* × *dt* is the entire physical recording time. We assume that these time steps are equidistant. Similar to Kobayashi et al. (2011), Paninski et al. (2012), and Lankarany et al. (2013b), the reversal potentials *E*_*L*_, *E*_*E*_, and *E*_*I*_, the leakage conductance *g*_*L*_, and the synaptic time constants τ_*E*_ and τ_*I*_ are known in our simulation studies. Note that the capacitance of the MP is set to 1 nF and therefore has been removed from Equation (1).

Our objective in this study is to develop a new algorithm, MtKF, to estimate the time course of excitatory and inhibitory SCs *g*_*E*_ and *g*_*I*_ for each trial during sensory stimulation, based on all repeated trials. Here, we assume that non-negative SIs are generated by a truncated Gaussian distribution. The probability distribution functions of the excitatory and inhibitory SIs are given by:

(2) $$\begin{array}{l} N_{E}(t)\;\overset{D}{=}\;Gauss\left(\mu_{E}(t),\;\Gamma_{E}(t)\right),N_{E}(t)\geq 0\\ N_{I}(t)\;\overset{D}{=}\;Gauss\left(\mu_{I}(t),\;\Gamma_{L}(t)\right),N_{I}(t)\geq 0 \end{array}$$

where μ_*E*_ (*t*) and μ_*I*_ (*t*) are the mean of the excitatory and inhibitory SIs, respectively, at time *t*, and Γ_*E*_ (*t*) and Γ_*I*_ (*t*) are the time-varying variances of these SIs at time *t*.

As shown in Lankarany et al. (2013b), estimations of *N*_*E*_ and *N*_*I*_ gives *g*_*E*_ and *g*_*I*_ directly according to Equation (1). It has been shown previously (Paninski et al., 2012; Lankarany et al., 2013b) that these SIs can be inferred from each single trial of the recorded MP using non-parametric statistical approaches. However, previous approaches required temporal smoothing of the estimates and therefore could not capture the fine-timescale fluctuations of the SIs (and SCs). To solve this issue, we propose a novel algorithm that takes advantage of all the recorded MP trials. This is achieved by applying an extended Kalman filter (EKF) (Lankarany et al., 2013b) to each trial, followed by an EM algorithm that calculates a *common* mean and variance of SIs [i.e., μ_*E*_(*t*), μ_*I*_(*t*), Γ_*E*_ (*t*), and Γ_*I*_ (*t*) in Equation (2)] from all recorded traces of the MP. Similar to the recursive framework in Lankarany et al. (2013b), these statistics are then used as the *a priori* knowledge for the EKF in the next iteration, and our algorithm repeats until no significant changes occur in the estimated dynamics.

### Proposed algorithm: multiple-trial kalman filtering (MtKF)

**Figure 1:**
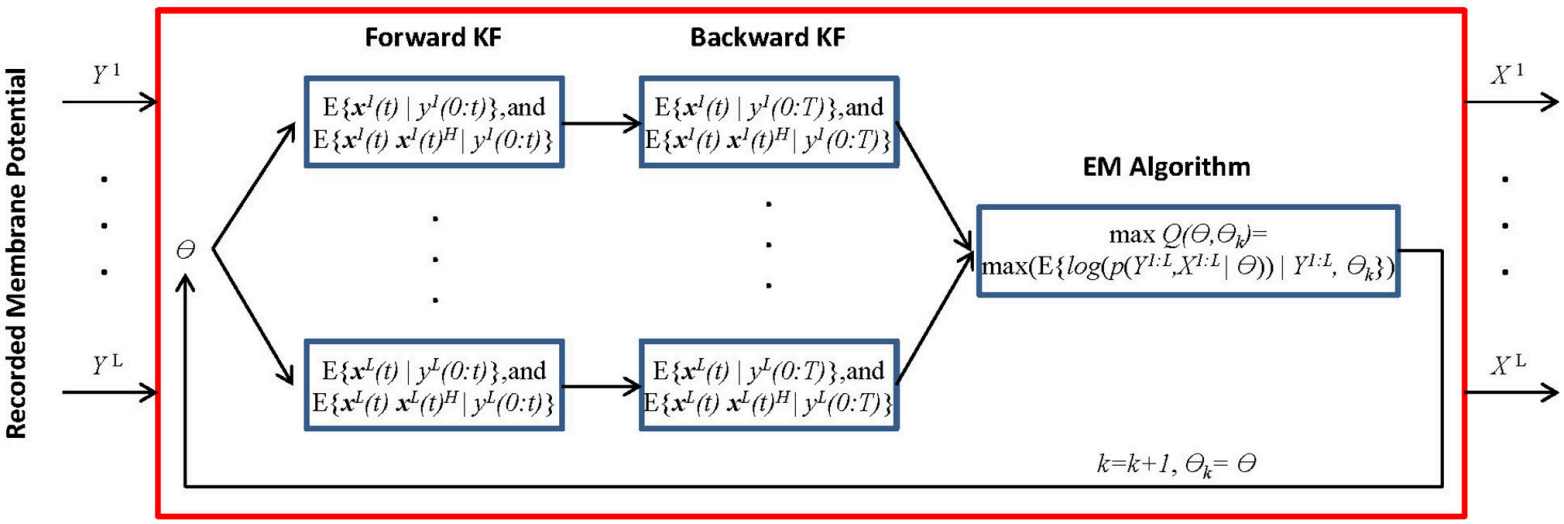
**Schematic representation of multiple-trial Kalman filtering (MtKF)**. The forward and backward KFs compute the system's state statistics based on parameter θ, and the following EM algorithm updates this θ. These processes are repeated until θ converges. ***x*** is the state of the system and *y* is the observation. Here, *k* and θ_0_ are the iteration number and the initial values of the statistical parameters, respectively. *X* and *Y* are abbreviations for the entire samples of ***x*** and *y* over time, i.e., *X* = ***x***(0:*T*) and *Y* = *y*(0:*T*). Index *i* indicates a trial index (*i* = 1, …,*L*) where *L* is the number of trials, θ is the unknown statistical parameters of the system and observation noise, θ = [μ_*E*_, μ_*E*_, Γ_*E*_, Γ_*I*_, σ_*V*_, σ_*Y*_], and (•)^H^ represents the matrix transpose operation.

We present the MtKF algorithm for identifying the excitatory and inhibitory SCs of a single neuron expressed by (Equation 1) from noisy MPs recorded during multiple stimulation trials. In the following sections, we use the notation *x*^*i*^(0:*t*) = {*x*^*i*^(0), *x*^*i*^(1), …, *x*^*i*^(*t*)} to represent the time trace of variable *x* = (*V, g*_*E*_, *g*_*I*_) corresponding to the *ith* recorded trial from time 0 to *t*. Figure 1 shows a block diagram of the MtKF algorithm.

The dynamical system underlying the generation of the MP in trial *i* is defined as

(3) $$\{\begin{array}{l} x^{i}(t+1)=F(x^{i}(t))+v(t)\\ y^{i}(t+1)=H(x^{i}(t+1))+\varepsilon^{i}(t) \end{array}$$

where **x**^*i*^(*t*) and *y*^*i*^(*t*) indicate the state vector (including the true MPs and SCs) and the observation (a noisy measurement of MP), respectively, of the *ith* trial at time *t*. Functions *F* and *H* are the transition and observation functions, respectively, and ***v***(*t*) and ε^*i*^ (*t*) are the system noise (comprising the common inputs) and the observation noise, respectively. An explicit form of these equations is introduced in the next section. While we assume above that the system is observed at each time step, it is easy to consider a case, in which observations are less frequent. In Figure 1, θ stands for the statistical parameters of ***v*** and ε, e.g., the mean and variances. Our objective is to estimate the dynamics of each trial (**x**^*i*^(*t*) *i* = {1, …, *L*}, where *L* is the number of trials) as well as to estimate the statistical parameters of the common synaptic input ***v***(*t*) and observation noise ε^*i*^(*t*). The MtKF algorithm begins with an arbitrary initiation followed by forward and backward Kalman filtering. These filtering steps are necessary to identify the hidden dynamics **x**^*i*^(*t*) for each trial given the noisy observation. Once this step is accomplished and the statistics (such as mean and variance) of the dynamics are calculated, the parameters of the common (shared) stochastic sources are inferred by using an EM algorithm. Since these parameters determine the initial values of the next iteration, the algorithm can be stopped with an appropriate criterion, i.e., no significant change in likelihood function in two consecutive iterations.

### Derivation of MtKF algorithm

We derive the MtKF algorithm in this section. Let ***x***(*t*) = [*V*^*i*^(*t*), $g_{E}^{i}$(*t*), $g_{I}^{i}$(*t*)]^H^ denote the vector of neuron dynamics corresponding to *ith* recorded voltage at time *t*, where [·]^H^ represents the matrix transpose operation. We can represent the neuronal model (Equation 1) in the dynamical-system form of Equation (3), where the observation function for each trial is given by *H*[***x***^*i*^(*t*)] = *C****x***^*i*^(*t*), with a vector *C* = [1, 0, 0], meaning that only the MP is observed. Similarly, the transition function *F* is given by

(4) $$\begin{array}{l} F[x^{i}(t)]=\left[\begin{array}{lll} 1-dt\left(g_{L}+g_{E}^{i}(t)+g_{I}^{i}(t)\right), & dt\;E_{E}, & dt\;E_{I}\\ \;0 & \;, & 1-\frac{dt}{\tau_{E}},0\\ \;0 & \;, & 0,\;1-\frac{dt}{\tau_{I}} \end{array}\right]\\ \;\left[\begin{array}{c} V^{i}(t)\\ g_{E}^{i}(t)\\ g_{I}^{i}(t) \end{array}\right]+\left[\begin{array}{c} dtg_{L}E_{L}\\ 0\\ 0 \end{array}\right] \end{array}$$

The distribution of the system noise (dynamical noise) ***v*** (*t*) = [*w* (*t*), *N*_*E*_(*t*), *N*_*I*_(*t*)]^H^ is given by

(5) $$\begin{array}{l} p(v_{t})=Gauss\left(\mu_{v}(t),\Gamma_{v}(t)\right),v_{t}\geq 0\\ \mu_{v}(t)=[0,\mu_{NE}(t),\mu_{NI}(t)]\&\Gamma_{v}(t)=\left[\begin{array}{c} \sigma_{w}^{2},\;0,0\\ 0,\;\Gamma_{NE}(t),\;0\\ 0,\;0,\;\Gamma_{NI}(t) \end{array}\right] \end{array}$$

where *N*_*E*_ and *N*_*I*_ describe excitatory and inhibitory SIs, respectively. Since we apply the EKF for each recorded MP (see Lankarany et al., 2013b for more details), we require to estimate the conditional probabilities *p*(***x***^*i*^(*t*)*|y*^*i*^(0:*t*)) and *p*(***x***^*i*^(*t*)*|y*^*i*^(0:*T*)) in the forward and backward KF, respectively (see Appendix I in Supplementary Material for details). In the next section, we derive the KF for each observed trial and calculate the statistical parameters of the common SIs using the EM algorithm.

#### Kalman forward/backward filtering

In a KF, we use a set of mathematical equations to estimate the present state of a dynamical system and employ the observed measurements to update its state accordingly (Haykin, 2001). In the EKF used here, the first-order Taylor linearization of the nonlinear process and measurement model is used to derive the underlying prediction–correction mechanism. Using Equation (1), the *a priori* state estimate and error covariance matrix are calculated at each time *t*. We then calculate the *a posteriori* state estimate and error covariance matrix for the same time instant. These variables are used in an iterative manner for the next time instant *t*+1, upon the arrival of a new observation. According to the proposed MtKF algorithm (Figure 1), we calculate the state estimate E{***x***^*i*^(*t*)|*y*^*i*^(0:*t*)} and state correlation matrix E{***x***^*i*^(*t*)***x***^*i*^(*t*)^H^|*y*^*i*^(0:*t*)} in the forward filtering step and E{***x***^*i*^(*t*)|*y*^*i*^(0:*T*)} and E{***x***^*i*^(*t*)***x***^*i*^(*t*)^H^|*y*^*i*^(0:*T*)} in the backward filtering (smoothing) step using the KF approach for each observed recorded MP. Here, E{·} stands for the expected value and ***x***_*i*_ is the state vector of the *ith* trial (see Appendix 2 and 3 of Lankarany et al., 2013b) for more details).

#### Inferring statistical parameters via expectation maximization

The EM algorithm is a robust optimization technique for inferring the parameters of models involving unobserved data (Dempster et al., 1977), e.g., the excitatory/inhibitory SIs *N*_*E*_(*t*) and *N*_*I*_(*t*) in this paper. We derive the EM algorithm to use all recorded traces of MP, and infer the statistics underlying common SIs. The EM algorithm infers the statistical parameters of Equations (3–5), i.e., the time-varying mean (μ_*v*_ (*t*)) and the variance of the states ($\sigma_{w}^{2}$, Γ_*v*_(*t*)), and the variance of the observation noise ($\sigma_{\varepsilon }^{2}$). We note that these statistics, for each excitatory and inhibitory SI, are shared over all recorded trials. Providing sufficient statistics of the state estimates (mean and correlation matrices) by Kalman filtering steps, we can easily derive the EM algorithm. To achieve this, we should maximize the logarithm of the joint probability of the states and observation (*X* and *Y* denote the entire samples of ***x*** and *y* over time, respectively) as follows:

(6) $$\begin{array}{l} \underset{s.t.\hat{\theta }}{\max Q(\theta ,\hat{\theta })}=\max(E\{\log\left(p(Y^{1},\ldots ,Y^{L},X^{1},\ldots ,X^{L}/\hat{\theta })\right)\\ \;/Y^{1},\ldots ,Y^{L},\theta \})\\ =\max\left(E\left\{\log\left(\prod_{i\;=\;1}^{L}p(Y^{i},X^{i}/\hat{\theta })\right)/Y^{1},\ldots ,Y^{L},\theta \right\}\right)\\ \;=\max\left(\sum_{i\;=\;1}^{L}\int \log\left(p(Y^{i},X^{i}/\hat{\theta })\right)p(X^{i}/Y^{i},\theta )dX^{i}\right) \end{array}$$

By doing the corresponding calculations to solve Equation (6) (as described in Appendix II in Supplementary Material), we can obtain the mean and variance of the common SIs (statistical parameters assigned to θ in Equation (5), i.e., the statistical parameters of the model, θ = [μ_*E*_, μ_*E*_, Γ_*E*_, Γ_*I*_, σ_*V*_, σ_*Y*_]) as well as the variance of the observation noise. As a result, we can update the statistical parameters of the excitatory and inhibitory SIs as well as the variance of the observation noise in the M-step (see Appendix II in Supplementary Material for full derivations). Inferring all parameters, we can initialize the next iteration of the recursive algorithm. The algorithm continues until there is no significant change (<1% increase in likelihood function) between two consecutive iterations.

Following the EM algorithm derived in Appendix II in Supplementary Material, the statistical parameters of the common SIs are estimated as follows:

(7) $$\begin{array}{l} \mu_{NE}(t)=\frac{1}{L}\sum_{i\;=\;1}^{L}\mu^{i}{}_{NE}(t)\\ \Gamma_{NE}(t)=\frac{1}{L}\sum_{i\;=\;1}^{L}\left\{\Gamma^{i}{}_{NE}(t)+{\left(\mu_{NE}(t)-\mu^{i}{}_{NE}(t)\right)}^{2}\right\} \end{array}$$

(8) $$\begin{array}{l} \mu_{NI}(t)=\frac{1}{L}\sum_{i\;=\;1}^{L}\mu^{i}{}_{NI}(t)\\ \Gamma_{NI}(t)=\frac{1}{L}\sum_{i\;=\;1}^{L}\left\{\Gamma^{i}{}_{NI}(t)+{\left(\mu_{NI}(t)-\mu^{i}{}_{NI}(t)\right)}^{2}\right\} \end{array}$$

Here, μ^*i*^_*NE*_, μ^*i*^_*NI*_, Γ^*i*^_*NE*_, and Γ^*i*^_*NI*_ are the mean (μ) and the variance (Γ) of the excitatory and inhibitory SIs corresponding to each single trial (see Appendix II in Supplementary Material). We repeat this update recursively within the EM algorithm until the estimates converge (see **Figure 3** for the manner in which the result converges with the number of iterations; see Supplementary Code for a sample code).

### Extension of multiple-trial framework for particle filtering (MtPF algorithm)

The proposed multiple–trial framework (Figure 1) is extended to the PF algorithm (Paninski et al., 2012). This approach is referred to as the multiple-trial PF (MtPF). Note that all the details about the derivation of PF for single-trial estimation of the excitatory and inhibitory SCs can be found in Paninski et al. (2012). Similar to the derivation of MtKF, we derive the EM algorithm to update the statistics of the common SIs, exploiting multiple recorded trials. As the first step, the algorithm runs parallel PF for individual trials to infer excitatory and inhibitory SCs $g_{E/I}^{i}\left(t\right)$ (*i* indicates the *i*^th^ trial) and the corresponding SIs ${\text{N}}_{\text{E/I}}^{\text{i}}\left(t\right)$, whose distributions are expressed as follows (exponential distribution as used in Paninski et al., 2012):

(9) $$\begin{array}{l} p\left[N_{E}^{i}(t)\right]=\frac{1}{\mu_{NE}(t)}\exp\text{​}\left(-N_{E}^{i}(t)/\mu_{NE}(t)\right)\\ p\left[N_{I}^{i}(t)\right]=\frac{1}{\mu_{NI}(t)}\exp\text{​}\left(-N_{I}^{i}(t)/\mu_{NI}(t)\right) \end{array}$$

Here, μ_*NE*_(*t*) and μ_*NI*_(*t*) are the trial means of the excitatory and inhibitory SIs, respectively. Similar to Equation (6), we wish to maximize the logarithm of the joint probability of the states and observation as follows:

(10) $$\begin{array}{l} \begin{array}{l} E_{p(N_{E},N_{I}/Y)}\left\{\sum_{i}\log\text{​}\left(p(y^{i},X^{i}/\hat{\theta })\right)/Y,\theta \right\}=\\ \sum_{i}\sum_{t\;=\;1}^{T}E_{p(N_{E},N_{I}/Y)}\{-\frac{1}{2}\log\sigma_{\varepsilon }^{2}+{\left(y^{i}(t)-V^{i}(t)\right)}^{H}{\left(\sigma_{\varepsilon }^{2}\right)}^{-1}\\ \;\left(y^{i}(t)-V^{i}(t)\right)\}+\\ \sum_{i}\sum_{t\;=\;1}^{T}E_{p(N_{E},N_{I}/Y)}\left\{\log{\left[\mu_{NE}(t)\right]}^{-1}-{\text{N}}_{E}^{i}(t){\left[\mu_{NE}(t)\right]}^{-1}\right\}+\\ \sum_{i}\sum_{t\;=\;1}^{T}E_{p(N_{E},N_{I}/Y)}\left\{\log{\left[\mu_{NI}(t)\right]}^{-1}-{\text{N}}_{I}^{i}(t){\left[\mu_{NI}(t)\right]}^{-1}\right\} \end{array} \end{array}$$

Then, given $E_{p\left(N_{E}/Y\right)}\left\{{\text{N}}_{E}^{i}\left(t\right)\right\}=\mu_{NE}^{i},E_{p\left(N_{I}/Y\right)}\left\{{\text{N}}_{I}^{i}\left(t\right)\right\}=\mu_{NI}^{i}$, the common statistics of the SIs are estimated as follows:

(11) $$\begin{array}{l} \mu_{NE}(t)=\frac{1}{L}\sum_{i\;=\;1}^{L}\mu_{\text{NE}}^{i}(t)\\ \mu_{NI}(t)=\frac{1}{L}\sum_{i\;=\;1}^{L}\mu_{\text{NI}}^{i}(t) \end{array}$$

It is clear from Equation (11) that the means of the SIs have the same update rule (only for mean SIs) as those presented in Equations (7) and (8). These means are then used for the next iteration of MtPF. Comparing the EM derivation of MtPF with that in Paninski et al. (2012), we see that the trial means of the SIs in MtPF are estimated using neither a nonlinear link function (an exponential function was used in Paninski et al., 2012) nor a non-parametric M-step that applies the B-spline method. Therefore, the closed-form update rule (EM algorithm) of MtPF is simpler than that in Paninski et al. (2012).

### Structured and non-structured synaptic inputs

**Figure 2:**
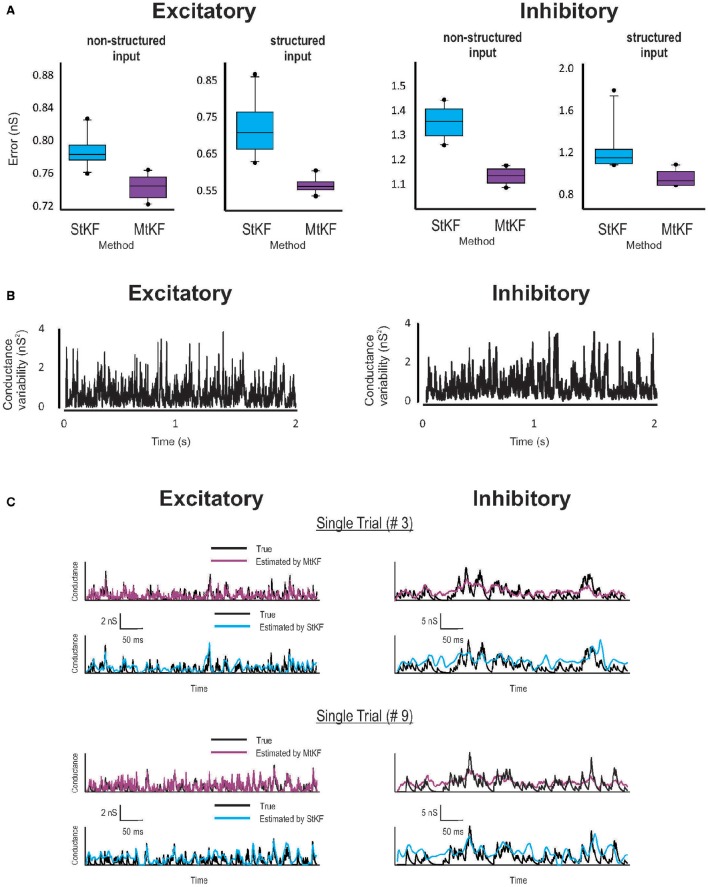
**Inferring Exc and Inh synaptic conductances (SCs) of the non-structured (OU process) and structured (periodic stimuli) inputs using MtKF and StKF. (A)** Error box for Exc (left) and Inh (right) SCs. Each box represents the RMSE calculated for 10 trials. **(B)** Trial-to-trial variability of Exc–Inh conductances, i.e., the time-dependent variance of those conductances. **(C)** Examples of time traces (single trials; two arbitrary trials are shown) of each algorithm vs. the original conductances. Color map, black (true); purple (MtKF), cyan (StKF).

The time-varying mean of the Poisson distribution for excitatory and inhibitory inputs is either structured or non-structured. A non-structured mean is modeled as an OU process (the absolute white noise (non-negative) is filtered based on excitatory and inhibitory time constants). To build a structured mean that mimics the post-synaptic potential (PSP) evoked by whisker stimulation (in our simulations), we used excitatory and inhibitory SCs of real neurons. Simulation results (those belonging to structured inputs) are shown for neuron #3 calculated using the least squares (LS) method (Heiss et al., 2008). These estimates are then smoothed and used as the time-varying mean in our simulations (Figures 2A, **4**). As a result, these traces (**Figure 4**) exhibit adaptation (Heiss et al., 2008) as seen in the *in vivo* data. We synthesize excitatory and inhibitory SIs, for both structured and non-structured inputs, from a Poisson distribution in our simulations. Excitatory and inhibitory SCs are then generated by Equation (1). We clarify that inferences by MtKF and MtPF are, respectively, based on Equations (2) and (9) in which the distributions of SIs are truncated Gaussian and exponential. In our simulations, we have generated SIs from Poisson distributions in order to evaluate the performances of those algorithms in non-ideal scenarios.

## Results

### Multiple-trial kalman filtering (MtKF) outperforms existing bayesian algorithms for simultaneously inferring excitatory and inhibitory synaptic conductances

The MtKF algorithm takes advantage of repeatedly recorded membrane-potential trials to better estimate the common excitatory and inhibitory SIs. Here, we show that the precise estimation of these common SIs leads to better inference of the excitatory and inhibitory SCs. We first simulate the membrane-potential dynamics with zero current injection to compare the performance of MtKF with that of StKF (Lankarany et al., 2013b). In all our simulations, the excitatory and inhibitory reversal potentials are E_E_ = 0 mV and E_I_ = −80 mV, respectively. The leak potential is E_L_ = −60 mV and the membrane time constant is 1/g_L_ = 12.5 ms. The excitatory and inhibitory synaptic time constants are τ_E_ = 3 ms and τ_I_ = 10 ms, respectively. The sampling time is 2 ms.

Examples are shown in Figure 2 for both *structured* mean SI (periodic stimuli; see Materials and Methods) and *non-structured* mean SI (random OU processes; see Materials and Methods). Given the common mean, the SIs in each trial (total *L* = 10 trials) are randomly generated by a Poisson distribution (see Materials and Methods). Synaptic conductances and the resulting MP trace are then generated according to Equation (1) and are continued for 2 s for each trial. A white Gaussian observation noise of standard deviation (std) 1 mV is added to the MP at each time step. The StKF and MtKF algorithms are then applied to infer the excitatory and inhibitory SCs from these membrane-potential traces. In order to quantify the trial-to-trial performances of each algorithm, we calculate the root-mean-square error (RMSE) between the true and estimated SCs for each single trial. The error bars are plotted in Figure 2A for both *non-structured* and *structured* SIs. Multiple-trial Kalman filtering outperforms StKF in estimating both excitatory and inhibitory SCs. The trial-to-trial variabilities of excitatory and inhibitory SCs are shown in Figure 2B. In order to show that MtKF improves the single-trial estimates (i.e., the ability to track the variabilities of each trial), two representative traces of arbitrarily selected single trials of excitatory and inhibitory SCs (for *non-structured* input) are plotted in Figure 2C. The conductances estimated by MtKF are better at tracking the true SCs in each trial (for inhibitory conductances, it is not as clear as excitatory conductances because of the weak driving force of inhibitory conductances for *I*_inj_ = 0; we suggest a better current level later).

**Figure 3:**
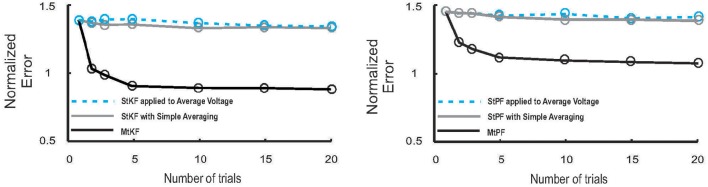
**Performance of MtKF and MtPF, unlike simple averaging of trials, improves when number of trials increases. Left:** normalized error (see Equation 12) of conductances is plotted against number of trials for MtKF (black) and StKF with simple averaging (gray) and StKF applied to the average of membrane potential (MP) (cyan). Note that the above-mentioned methods would have the same performance if such variability is negligible over trials. **Right:** normalized error (same as left) for MtPF (black) and StPF with simple averaging (gray) and StPF applied to the average of MP (cyan).

We note that StKF and MtKF are equivalent if only one trial is applied. The main advantage of our proposed MtKF algorithm is that it uses all recorded trials to estimate the statistics of the common SI and therefore is better at inferring the SCs in each single trial. In order to highlight this advantage of MtKF and to demonstrate that our algorithm is not just a simple averaging of StKF, the normalized error of the SCs is plotted against the number of trials (for zero injected current) in Figure 3 for three different methods: MtKF, the average of the StKF method (the average conductance estimated by StKF applied to each trial), and the StKF method applied to the average MP. The normalized error is defined as

(12) $$\bar{Err}=\frac{\left(\bar{Err_{E}}+\bar{Err_{I}}\right)}{2},$$

where ${\bar{Err}}_{E/I}=\sqrt{\frac{1}{T}\sum_{t}\frac{\mathrm{var}\left(g_{E/I}\left(t\right)-{ĝ}_{E/I}\left(t\right)\right)}{\mathrm{var}\left(g_{E/I}\left(t\right)\right)}}$, var calculates the trial variance, and ĝ_*E*/*I*_ describes the estimated SC. This normalized error weights the error of excitatory and inhibitory SCs with the same contribution. As we can see from Figure 3 (left), the normalized error for MtKF decreases when the number of trials increases, whereas this error remains approximately constant for the other two methods. This suggests that those methods do not benefit fully from multiple trials for estimating single-trial SCs. Instead, they can only estimate the trial average of excitatory and inhibitory SCs. The key consideration in this example is the trial-to-trial variability of SCs. If such variability is small, all mentioned methods perform effectively equivalently. However, if the variability over trials is high, simple averaging methods cannot track the changes of single trials, whereas MtKF benefits from all trials to better estimate the SCs of the individual trials.

### Multiple-trial particle filtering (MtPF): multiple-trial framework is generalizable

Consistent with Figure 3 (left), we also demonstrate that MtPF outperforms the simple averaging methods based on single-trial particle filtering (StPF) (Paninski et al., 2012) for estimating excitatory and inhibitory SCs of individual trials. In particular, we compare the performances of MtPF, the average of StPF, and StPF applied to the average of MP. Figure 3 (right) shows that the simple averaging methods do not benefit from multiple trials (approximately constant normalized error for different numbers of trials) whereas the normalized error decreases for MtPF when the number of trials is increased. Hence, the multiple-trial EM framework that we propose is generalizable to different kinds of single-trial Bayesian methods.

### Improvement of the estimated excitatory and inhibitory synaptic conductances by optimizing the level of current injection

**Figure 4:**
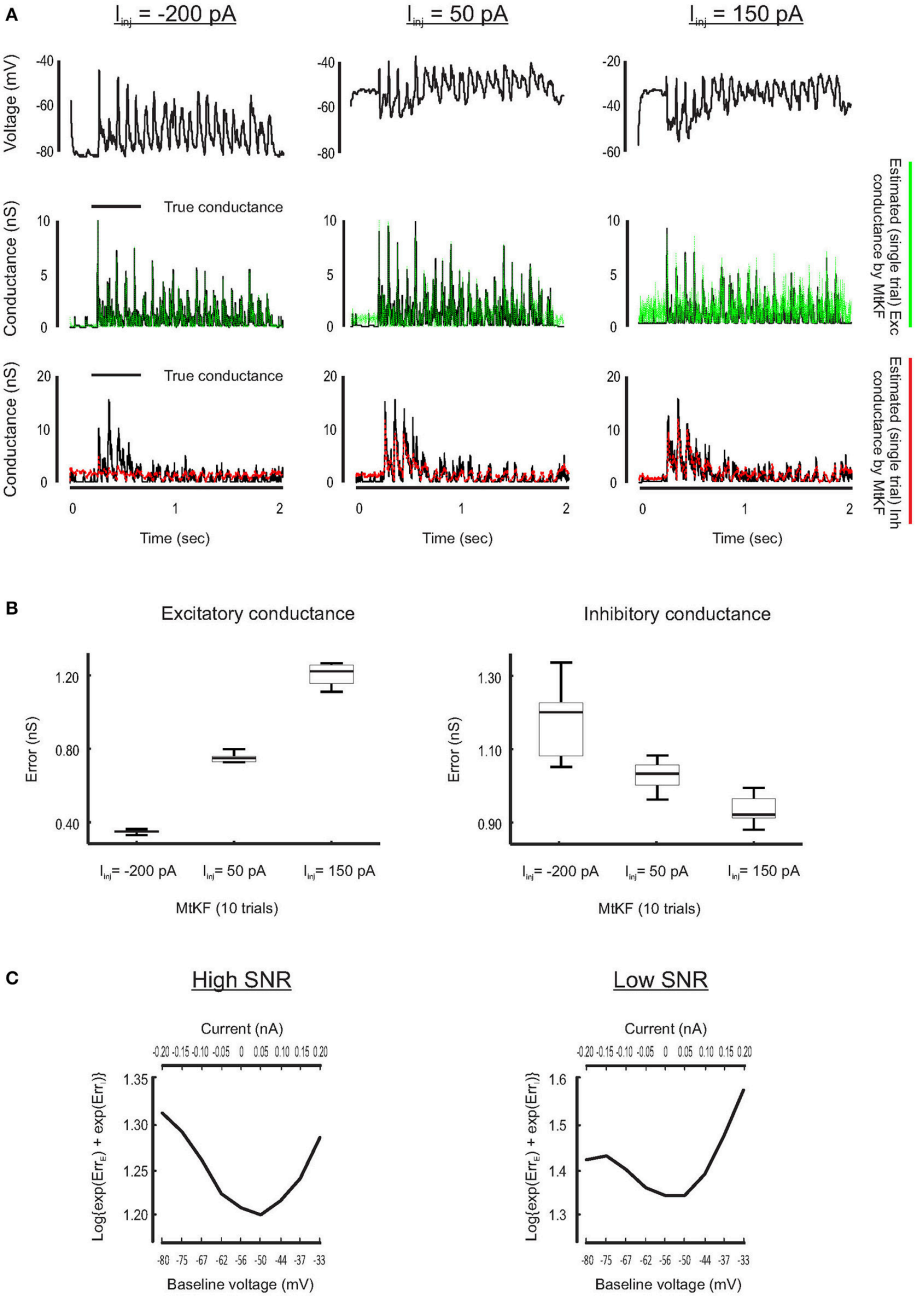
**Finding the best level of injected current, in current-clamp recording, for estimating the Exc and Inh SCs at individual trials. (A)** Examples of the Exc–Inh estimates for three different values of injected current, namely, I_inj_ = −200 pA (left), I_inj_ = 50 pA (middle) and the I_inj_ = 150 pA (right). **(B)** Error box for excitatory and inhibitory SCs including all trials. Each box represents the RMSE calculated for 10 trials. **(C)** The total error of excitatory and inhibitory conductances (from their original values) estimated by MtKF algorithm for high (left: std of observation noise is 0.1 mV) and low (right: std of observation noise is 1 mV) SNRs. The total error is calculated as the log{exp(*Err*_*E*_) + exp(*Err*_*I*_)}, where *Err*_*E*/*I*_ were already defined. The total error is plotted against the baseline level of MP (bottom axis) and the level of injected current (top axis). As can be seen, the total error has a minimum for I_inj_ = 50 pA (baseline = −50 mV).

Inference of SC is less accurate if the MP is close to its reversal potential because the synaptic driving force is small there. For example, this typically happens for the inhibitory conductance when no current is injected to a recorded neuron but a Bayesian approach is applied nonetheless to simultaneously estimate excitatory and inhibitory SCs (see Figure 1). To visually compare how the SCs are estimated through different levels of the injected currents, we plot a single trial of true excitatory and inhibitory SCs against the corresponding estimated SCs (*L* = 10 trials) for three different injected currents, I_inj_ = [−200, 50, 150] pA. Each current level is simulated by adding a constant current to the right-hand side of the MP dynamics of Equation (1). As can be observed from Figure 4A, both excitatory and inhibitory conductances are estimated with good resolutions for I_inj_ = 50 pA (inhibitory conductance is underestimated for I_inj_ = −200 pA, whereas excitatory conductance is very noisy for I_inj_ = 150 pA).

Furthermore, we quantify the performance of the proposed MtKF algorithm at estimating excitatory and inhibitory SCs at the above holding currents. The RMSE between true and estimated conductances is calculated for each trial (*L* = 10 trials corresponding to each level of current) and presented as an error box. Figure 4B shows that there is a trade-off between the accuracies of the estimated excitatory and inhibitory SCs. Our next objective is to find an optimum level of injected current that provides a balance between the accuracies of the estimated excitatory and inhibitory SCs such that both conductances are estimated reasonably accurately. To meet this objective, we investigate in our simulation study which level of injected current results in the most accurate estimation of both excitatory and inhibitory SCs. Through several trials, different levels of current are injected to the model neuron and the MP traces are calculated. We apply the proposed MtKF algorithm to the MP traces that are recorded with each level of injected current. We define a new error measurement that capitalizes the greatest of the excitatory and inhibitory errors, i.e., $\log\left\{\exp\left({\bar{Err}}_{E}\right)+\exp\left({\bar{Err}}_{E}\right)\right\}$, where ${\bar{Err}}_{E}$ and ${\bar{Err}}_{I}$ are defined in Equation (12). We run the MtKF algorithm in this simulation setup for low and high signal-to-noise ratios (SNRs) (observation noise with std of 1 mV for low SNR, and that of 0.1 mV for high SNR). Figure 4C shows that this error measure is smallest at the baseline MP around −50 mV (I_inj_ = 50 pA) for both SNRs. Therefore, we suggest applying the MtKF algorithm with this best level of the injected current as a new technique. This way, excitatory and inhibitory SCs can be accurately estimated simultaneously using a single level of current injection in the current-clamp recordings.

### Inferring excitatory and inhibitory synaptic conductances from *in vivo* recordings

**Figure 5:**
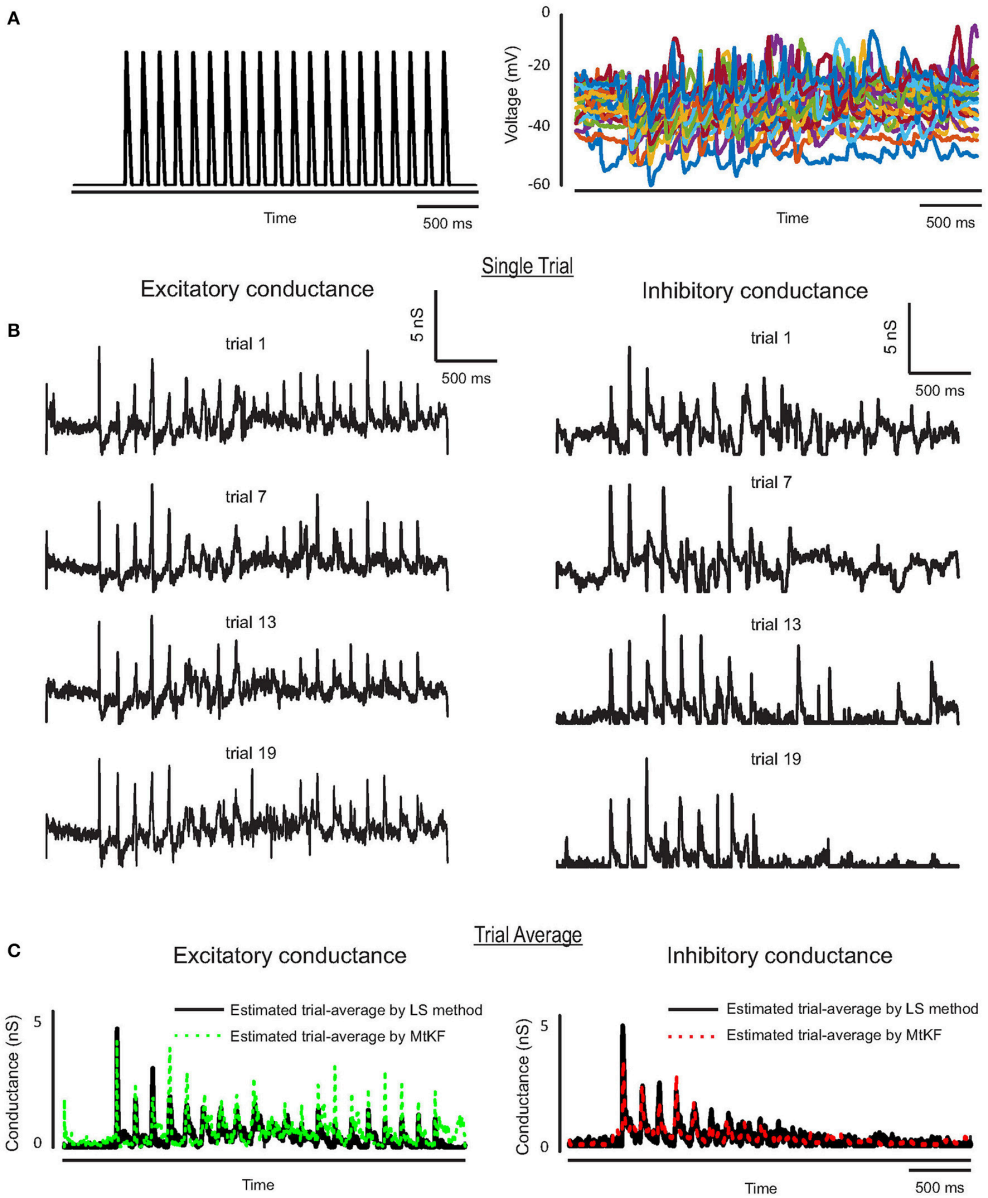
**(A)** Whisker stimulus (left) and recorded MP (25 trials) from I_inj_ = 130 pA in current-clamp mode. **(B)** Estimated single trials (four arbitrary trials are selected) of excitatory and inhibitory SCs, trials (from top to bottom) #16, 13, 3, 7. **(C)** Trial average of estimated conductances using MtKF algorithm (colors) vs. that estimated by least-squares (LS) method (black) using all different levels of injected currents.

**Figure 6:**
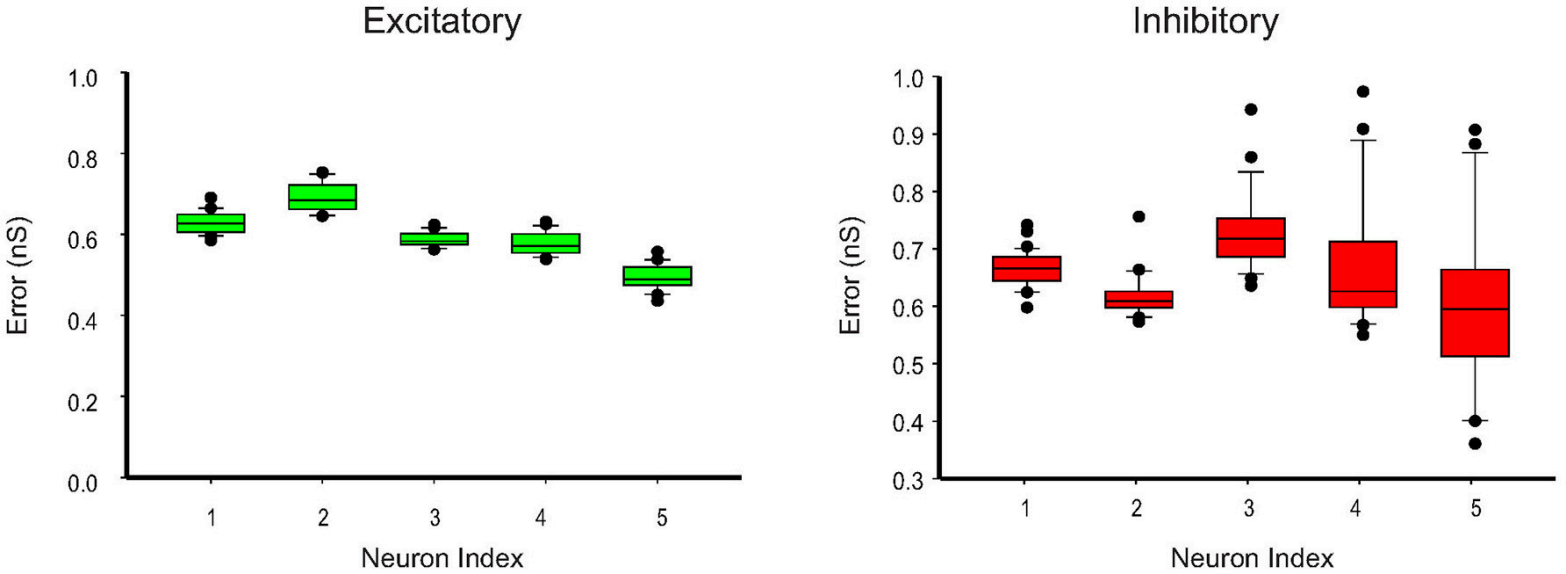
**Error box for excitatory and inhibitory SCs including all trials (25 trials corresponding to I_inj_ = 130 pA)**. The root-mean-square error (RMSE) between the estimated conductances, for each trial, and the trial average (calculated by LS method) is shown.

We further apply the MtKF algorithm to estimate the single trials of SCs from single neurons (*n* = 5) responding to whisker stimulation in the rat barrel cortex. As mentioned before, all details about the recording can be found in Heiss et al. (2008). In accordance with our results in the previous section, we apply the MtKF algorithm to membrane-potential traces with a fixed level of current injection (I_inj_ = 130 pA, baseline MP is about −35 mV). We note that although four different holding potentials were available in Heiss et al. (2008), the baseline of sub-threshold MP of I_inj_ = 130 pA was the closest to that of the optimal one (Figure 4C). Figure 5A shows the whisker stimulation pulses and 35 trials of recorded membrane-potential traces, from which we infer SCs. Figure 5B shows the estimated conductances of neuron #1 for four arbitrarily selected trials. Since the actual values of these conductances are unknown, and in order to verify the accuracy of our estimates, we compare the trial average of conductances estimated by MtKF with that obtained by the standard least squares (LS) method (Heiss et al., 2008). Note that four different levels of injected current (total ≥120 trials) are used for LS, whereas only one level of injected current (25 trials) is used for MtKF. Figure 5C compares trial-averaged traces of excitatory and inhibitory SCs estimated by the MtKF and LS methods. Figure 6 summarizes the RMSE of MtKF and LS as a proxy for the error for both excitatory and inhibitory conductances for all neurons. These results show that the estimated trial averages of the excitatory and inhibitory SCs are very close to those calculated by the LS method. This confirms the practicality of our methodology.

## Discussion

We proposed a new multiple-trial EM framework to simultaneously infer the excitatory and inhibitory SCs in individual trials from the recorded MP. This method inherits the general advantage of Bayesian approaches (e.g., single-trial PF, Paninski et al., 2012 and KF Lankarany et al., 2013b) that can optimize underlying statistics of the SI according to data. This is in contrast to previous non-Bayesian techniques that require some parameter pre-settings about oversampling period or slow/fast timescale separation (Bédard et al., 2012; Berg and Ditlevsen, 2013). More importantly, unlike previous single-trial Bayesian methods, this framework can optimize common SI statistics without requiring temporal smoothing steps by utilizing all recorded trials of the MP. Extending previous single-trial Bayesian methods under this framework, we developed multiple-trial Kalman filtering (MtKF) and particle filtering (MtPF). We showed that MtKF and MtPF provide superior performance relative to the corresponding single-trial Bayesian method that can use only one trial of MP at a time. The advantage becomes increasingly evident as the number of available trials increases (Figure 3). This technique is readily applicable to existing MP data, where a single level of current injection was applied, to estimate both excitatory and inhibitory conductances in each trial.

The relative estimation error of excitatory and inhibitory SCs is known to depend on the level of current injection. We applied the MtKF algorithm to quantify how the level of current injection affects simultaneous estimation of excitatory and inhibitory SCs (Figure 4). Our numerical simulations showed that the optimal level of injected current is robust to observation noise. Hence, the MtKF algorithm with this optimal current can reliably estimate both excitatory and inhibitory SCs in individual trials.

We also applied the MtKF algorithm to *in vivo* intracellular data from the rat barrel cortex. Using the near-to-optimum level of injected current, we simultaneously inferred excitatory and inhibitory SCs in each trial. The accuracy of our estimates was confirmed by comparing the trial average results with those from the conventional LS technique (where multiple levels of current injection are used). The estimation results of MtKF that used a single level of current injection matched well with those of the LS technique that required multiple levels of current injection.

An alternative approach can be used to extract specific joint statistics between excitatory and inhibitory SCs. For example, Tan et al. (2013) proposed a methodology to calculate the Pearson correlation of excitatory and inhibitory SCs from stationary data based on the dependency of the variance of membrane current on holding potential. This approach is distinct from MtKF because MtKF infers the full time course of conductances as opposed to only certain statistics. Secondly, while this approach requires recordings at multiple holding potentials, MtKF can simultaneously infer excitatory and inhibitory conductances by applying a single level of current injection. Furthermore, the ability of MtKF to efficiently integrate observations at neighboring time points is an additional benefit for analyzing non-stationary data.

In this study, to improve the estimation performance of MtKF, we utilized multiple recording trials with the same stimulation protocol. However, application of the stimulus is not a strict requirement for MtKF to benefit from multiple recording trials. Improvement should be observed as long as excitatory or inhibitory SI is correlated across trials. In this sense, multiple recording trials without any stimulation should also benefit the estimation because some statistics about SI are expected to be common across trials. Obviously, additional information that can better align SIs across different trials would allow more efficient extraction of common underlying features about SIs. This could be, for example, motor actions, onsets of up/down state transitions (Shu et al., 2003), or oscillation phases of local field potential (Buzsáki and Draguhn, 2004).

One limitation of MtKF is that it assumes a Gaussian distribution of SIs. If this assumption is invalid (e.g., if the distribution is characterized by a long tail or is bimodal), the method would not work so well. One approach to resolve this issue is to align trials precisely so as to minimize the variability of trials. As described above, if the distribution of SI is bimodal because of up and down states (Katz et al., 2006), that bimodal variability could be removed by aligning trials according to an up/down transition. Another approach is to apply the multiple-trial EM framework to Gaussian mixture Kalman filtering (Lankarany et al., 2013b), where the distribution of SIs is characterized by a Gaussian mixture model. A drawback of this approach is that it would require additional computational complexity. Hence, the complexity of the data should be appropriately chosen considering the trade-off between accuracy and computational complexity for a given amount of data.

In summary, the general applicability of the multiple-trial EM framework and the promising results of MtKF and MtPF obtained from both the synthetic and *in vivo* data suggest that this framework is capable of extracting the intricate interplay between excitatory and inhibitory SCs underlying an animal's neuronal activity.

## Author contributions

ML, TT developed algorithm. JH, IL recorded and provided *in-vivo* data. ML, JH, IL, and TT wrote the paper.

### Conflict of interest statement

The authors declare that the research was conducted in the absence of any commercial or financial relationships that could be construed as a potential conflict of interest.
