# Supplementary material for: Simultaneous Bayesian Estimation of Excitatory and Inhibitory Synaptic Conductances by Exploiting Multiple Recorded Trials
Source: Front Comput Neurosci. 2016 Nov 4;10:110. doi: 10.3389/fncom.2016.00110 (PMC5095134; doi:10.3389/fncom.2016.00110)
Supplement: Supplementary file 1 [file SupplementaryMaterial.pdf]

Supplementary Material

Simultaneous Bayesian Estimation of Excitatory and Inhibitory Synaptic Conductances by Exploiting Multiple Recorded Trials

Milad Lankarany, Jaime E. Heiss, Ilan Lampl and Taro Toyoizumi

Contents

Appendix I. Problem Formulation

Appendix II. Inference of statistical parameters

Appendix I. Problem Formulation

The dynamical system representing the dynamics of each recorded membrane potential (MP) is defined as follows:

$$\begin{cases} \mathbf{x}^{\mathrm{i}}(t+1) = f(\mathbf{x}^{\mathrm{i}}(t)) + \mathbf{v}(t) \\ y^{i}(t+1) = \hat{C}\mathbf{x}^{\mathrm{i}}(t+1) + \sqrt{dt}\,\sigma_{y}\varepsilon^{i}(t) \end{cases}, \tag{A.1}$$

where $\boldsymbol{v}(t)=[w(t), N_E(t), N_I(t)]^H$, index i corresponds to the ith Kalman filter that is derived for the ith trial, and

$$\begin{aligned} &\mathbf{x}^{i}(t) = [V^{i}(t), g_{E}^{i}(t), g_{I}^{i}(t)]^{H} \\ &\mathrm{X} = [\mathbf{x}^{1}(0:T),\ldots,\mathbf{x}^{i}(0:T),\ldots,\mathbf{x}^{L}(0:T)] \end{aligned}. \tag{A.2}$$

Both excitatory and inhibitory synaptic inputs (SIs) are approximated by a Gaussian distribution:

$$\begin{aligned} &\boldsymbol{N_E}(t) \approx Gaussian\,(\boldsymbol{N_E}(t); \boldsymbol{\mu}_{NE}(t), \boldsymbol{\Gamma}_{NE}(t)), \\ &\boldsymbol{N_I}(t) \approx Gaussian\,(\boldsymbol{N_I}(t); \boldsymbol{\mu}_{NI}(t), \boldsymbol{\Gamma}_{NI}(t)) \end{aligned} \tag{A.3}$$

At each iteration, the algorithm runs L Kalman filters independently in order to estimate the $\boldsymbol{x}^{\mathrm{i}}(1:T)$, for $i=1,\ldots, L$. Given all the $\boldsymbol{x}^{\mathrm{i}}(1:T)$, we aim to infer the mean and variance of the $N_E(1:T)$ and $N_I(1:T)$. The algorithm iterates using the estimated mean and variance of these SIs.

Appendix II. Inference of statistical parameters

We run Kalman filters (see details in Appendix 4 of [1]) for L trials to compute sufficient statistics required for EM algorithm.

We derive EM for our problem as described below.

$$
\begin{aligned}
&E_{p(N_E,N_I/Y)}\left\{\sum_i \log\Big(p(y^i, X^i/\hat{\theta})\Big)/Y,\theta\right\} = \\
&\sum_i\sum_{t=1}^{T} E_{p(N_E,N_I/Y)}\left\{-\frac{1}{2}\log\sigma_\varepsilon^2 + \big(y^i(t)-V^i(t)\big)^H \big(\sigma_\varepsilon^2\big)^{-1}\big(y^i(t)-V^i(t)\big)\right\} + \\
&\sum_i\sum_{t=1}^{T} E_{p(N_E,N_I/Y)}\left\{-\frac{1}{2}\log\Gamma_{NE}(t) + \big(\mathrm{N}_E^i(t)-\mu_{NE}(t)\big)^H \big(\Gamma_{NE}(t)\big)^{-1}\big(\mathrm{N}_E^i(t)-\mu_{NE}(t)\big)\right\} + \\
&\sum_i\sum_{t=1}^{T} E_{p(N_E,N_I/Y)}\left\{-\frac{1}{2}\log\Gamma_{NI}(t) + \big(\mathrm{N}_I^i(t)-\mu_{NI}(t)\big)^H \big(\Gamma_{NI}(t)\big)^{-1}\big(\mathrm{N}_I^i(t)-\mu_{NI}(t)\big)\right\}
\end{aligned}
\qquad \text{(A.4)}
$$

Note that we aim to estimate a common SI for all given trials, where the dynamics of each trial produce their own synaptic perturbation (mean and variance of SIs underlying each single trial) as expressed below.

$$
\begin{aligned}
N_E^i(t) &= \mu_{NE}^i(t) + \sqrt{\Gamma_{NE}^i(t)}\,\xi_E^i(t),\ \ \xi_E^i(t) = Gauss(0,1) \\
N_I^i(t) &= \mu_{NI}^i(t) + \sqrt{\Gamma_{NI}^i(t)}\,\xi_I^i(t)\ ,\ \ \xi_I^i(t) = Gauss(0,1)
\end{aligned}
\qquad \text{(A.5)}
$$

The terms $\mu^i{}_{NE}$, $\mu^i{}_{NI}$, $\Gamma^i{}_{NE}$, and $\Gamma^i{}_{NI}$ indicate the conditional mean (μ) and variance (Γ) of the SI given the observation of the ith trial, namely, $\mu_{N_{E,I}}^i = E\{\mathrm{N}_{E,I}^i(t)\,|\,Y^i\}$ and $\Gamma_{N_{E,I}}^i = \mathrm{var}\{\mathrm{N}_{E,I}^i(t)\,|\,Y^i\}$. Note that we use (A.5) to simply express the conditional probabilities of SIs in (A.4).

By taking the derivative of (A.4) with respect to μ_N and Γ_N (for both excitatory and inhibitory inputs), the EM algorithm results in estimating the statistical parameters of the common SIs as follows:

$$
\begin{aligned}
\mu_{NE}(t) &= \frac{1}{L}\sum_{i=1}^{L} \mu^i{}_{NE}(t) \\
\Gamma_{NE}(t) &= \frac{1}{L}\sum_{i=1}^{L} \{\Gamma^i{}_{NE}(t) + (\mu_{NE}(t) - \mu^i{}_{NE}(t))^2\}
\end{aligned}
\qquad \text{(A.6)}
$$

$$
\begin{aligned}
\mu_{NI}(t) &= \frac{1}{L}\sum_{i=1}^{L} \mu^i{}_{NI}(t) \\
\Gamma_{NI}(t) &= \frac{1}{L}\sum_{i=1}^{L} \{\Gamma^i{}_{NI}(t) + (\mu_{NI}(t) - \mu^i{}_{NI}(t))^2\}
\end{aligned}
\qquad \text{(A.7)}
$$

To finish our EM section, we calculate, for excitatory and inhibitory SIs, the conditional mean of each trial, i.e., $\mu^i{}_{NE}$ and $\mu^i{}_{NI}$, and the trial average of the conditional variances, i.e., $\frac{1}{L}\sum_{i=1}^{L} \Gamma^i{}_{NE}$

and $\frac{1}{L}\sum_{i=1}^{L} \Gamma^{i}{}_{NI}$. Note that all sufficient (conditional) statistics of SCs have been calculated previously by KF.

Consider the dynamics of SCs in (1):

$$g_{E,I}(t+1) = (1 - dt/\tau_{E,I})g_{E,I}(t) + N_{E,I}(t) \tag{A.8}$$

The mean and variance of SCs can be calculated as follows (see [2] for more details):

$$\begin{aligned} &E\{g_{E,I}(t)\} = (1 - dt/\tau)E\{g_{E,I}(t-1)\} + E\{N_{E,I}(t-1)\} \\ &\mathrm{var}\{g_{E,I}(t)\} = (1 - 2dt/\tau)\,\mathrm{var}\{g_{E,I}(t-1)\} + \mathrm{var}\{N(t-1)\} \end{aligned} . \tag{A.9}$$

Using the generic form of (A.9) for our problem, we have

$$\begin{aligned} &\mu^{i}{}_{NE}(t) = E\{g_E^i(t+1)\,|\,Y^i\} - (1 - dt/\tau_E)\,\mathrm{E}\{g_E^i(t)\,|\,Y^i\} \\ &\mu^{i}{}_{NI}(t) = E\{g_I^i(t+1)\,|\,Y^i\} - (1 - dt/\tau_I)\,\mathrm{E}\{g_I^i(t)\,|\,Y^i\} \\ &\frac{1}{L}\sum_{i=1}^{L} \Gamma^{i}{}_{NE}(t) \approx \frac{1}{L}\sum_{i=1}^{L} \left[\mathrm{var}\{g_E^i(t+1)\,|\,Y^i\} - (1 - 2dt/\tau_E)\ \mathrm{var}\{g_E^i(t)\,|\,Y^i\}\right] \\ &\frac{1}{L}\sum_{i=1}^{L} \Gamma^{i}{}_{NI}(t) \approx \frac{1}{L}\sum_{i=1}^{L} \left[\mathrm{var}\{g_I^i(t+1)\,|\,Y^i\} - (1 - 2dt/\tau_I)\ \mathrm{var}\{g_I^i(t)\,|\,Y^i\}\right] \end{aligned} \tag{A.10}$$

The approximations in the previous two equations become more accurate if the number of trials L is large. Again, note that the statistics on the right-hand sides of these equations are calculated by KF.

References

[1] Lankarany, M., Zhu, W. P., Swamy, M. N., and Toyoizumi, T. (2013). Inferring trial-to-trial excitatory and inhibitory synaptic inputs from membrane potential using Gaussian mixture Kalman filtering. Front. Comput. Neurosci. 7, 109. doi: 10.3389/fncom.2013.00109

[2] Gillespie, D. T. (1996). The mathematics of Brownian Motion and Johnson noise. Am. J. Phys. 64, 225–240.
